# *In-situ* tumor vaccination by percutaneous ablative therapy and its synergy with immunotherapeutics: An update on combination therapy

**DOI:** 10.3389/fimmu.2023.1118845

**Published:** 2023-03-08

**Authors:** Nicole J. Kim, Jessica H. Yoon, Adam C. Tuomi, John Lee, Daehee Kim

**Affiliations:** ^1^ Warren Alpert Medical School of Brown University, Providence, RI, United States; ^2^ Department of Diagnostic Imaging, Warren Alpert Medical School of Brown University, Providence, RI, United States

**Keywords:** tumor microenvironment, immunogenicity, percutaneous ablation, antitumor immunity, tumor neoantigen

## Abstract

Percutaneous tumor ablation is now a widely accepted minimally invasive local treatment option offered by interventional radiology and applied to various organs and tumor histology types. It utilizes extreme temperatures to achieve irreversible cellular injury, where ablated tumor interacts with surrounding tissue and host *via* tissue remodeling and inflammation, clinically manifesting as post-ablation syndrome. During this process, *in-situ* tumor vaccination occurs, in which tumor neoantigens are released from ablated tissue and can prime one’s immune system which would favorably affect both local and remote site disease control. Although successful in priming the immune system, this rarely turns into clinical benefits for local and systemic tumor control due to intrinsic negative immune modulation of the tumor microenvironment. A combination of ablation and immunotherapy has been employed to overcome these and has shown promising preliminary results of synergistic effect without significantly increased risk profiles. The aim of this article is to review the evidence on post-ablation immune response and its synergy with systemic immunotherapies.

## Introduction

Interventional radiology is an essential part of oncologic care, from its traditional roles in tissue sampling and venous access to active therapeutics in tumor-directed locoregional therapies and various palliative interventions. In addition, recent progress in immunotherapy brought particular interest to systemic and local tumor microenvironments created by locoregional interventional therapies given their potential to release tumor-specific neoantigen and other immune-stimulators *via in-situ* tumor destruction (1, 2).

Percutaneous tumor ablation is now a widely accepted minimally invasive local treatment option offered by interventional radiology and applied to various organs and tumor histology types (3, 4). Tissue ablative technologies utilize extreme temperatures to achieve irreversible cellular injury (3). Radiofrequency ablation (RFA) has the longest history of clinical utilization and there are now various other modalities including microwave ablation (MWA), cryoablation (CA), high-intensity focused ultrasound (HIFU), and laser ablation (LA). Another tissue ablation modality, Irreversible electroporation (IRE), differs from others as it induces non-thermal cellular injury. Regardless of specific modalities, ablated tumor interacts with surrounding tissue and host *via* tissue remodeling and inflammation. Clinically, this is characterized as a post-ablation syndrome, which manifests as a combination of flu-like symptoms such as fever, malaise, nausea, and others (5–7). During this process, tumor neoantigens are released from ablated tissue and prime one’s immune system which would favorably affect both local and remote site disease control (so called “abscopal effect”). Expectedly, there has been a strong interest in taking advantage of this phenomenon, especially in the form of a combination between ablation and immunotherapy. The aim of this article is to review the evidence on post-ablation immune response and its synergy with systemic immunotherapies.

## Cellular injury and tumor neoantigen release after percutaneous ablative therapy

**Figure 1:**
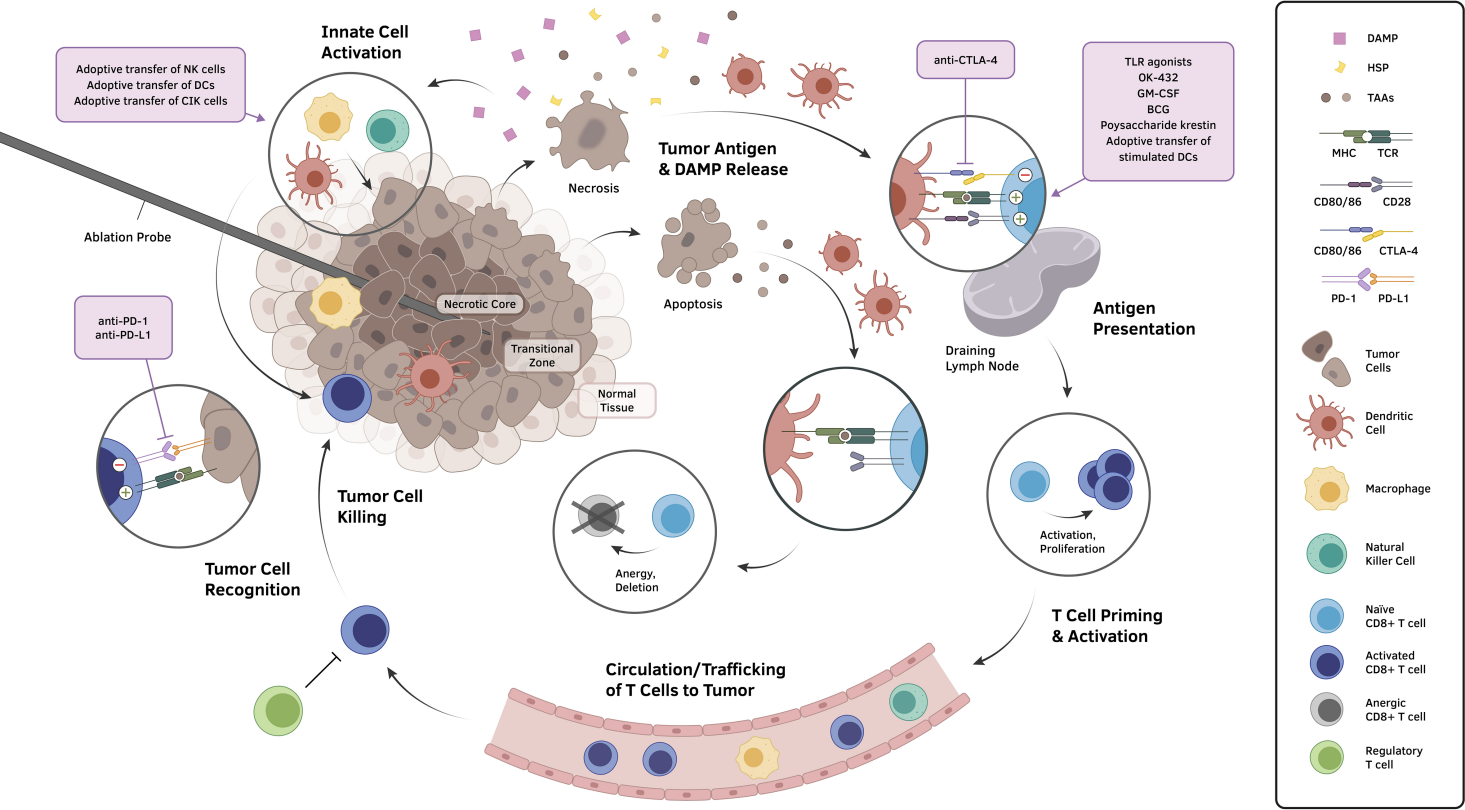
The application of ablative therapeutic modalities to tumors produces a biomolecular landscape that can generally be conceptualized into three concentric zones: a central necrotic zone, a transitional zone demonstrating the consequences of varying degrees of cellular injury, and an outer zone of normal tissue. Cells undergoing necrosis and other non-apoptotic forms of regulated cell death expose and release an abundance of molecules, including TAAs, tumor neoantigens, HSPs, and DAMPs, that incur the recruitment of innate cells, activation of local effector cells, and maturation of antigen presenting cells, particularly DCs. These DCs migrate to draining lymph nodes and induce the formation of tumor-specific cytotoxic lymphocytes, which subsequently circulate to the tumor site and carry out killing of tumor cells upon target antigen recognition. However, multiple negative immunomodulatory mechanisms that exist throughout the cycle, such as T cell anergy resulting from apoptotic tumor cell signaling and the induction of immunosuppressive phenotypes in the tumor microenvironment, have hindered clinically meaningful, immunologically driven anticancer effects from manifesting following ablation. Various approaches to overcome such immunosuppressive obstacles (violet boxes) have been deployed in conjunction with locoregional ablation to demonstrate prospects for a synergistic reinstatement of an immunostimulatory anti-tumor activities. Abbreviations: DAMP, damage-associated molecular pattern; DC, dendritic cell; HSP, heat shock protein; MHC, major histocompatibility complex; TAA, tumor-associated antigen; TCR, T cell receptor; TLR, toll-like receptor.

Percutaneous ablative therapies, except for IRE, induce cellular injury by exposing tumor tissue to extreme temperatures. RFA, MWA, HIFU, and LA induce hyperthermic cellular injury, while the lethal freezing temperature is used in cryoablation. Focally induced extreme temperature results in a spherical area characterized by three distinct zones: central necrotic, peripheral transitional, and normal tissue (1, 3, 4, 8) (**Figure 1**). The central necrotic zone is immediately adjacent to ablation probes, achieved by temperatures greater than 60°C or below -20°C (3, 8), resulting in direct and immediate cellular injury. On the other hand, the peripheral transitional zone is characterized by a band of thermal conduction between the central necrotic zone and surrounding normal tissue. A steep temperature gradient within the transitional zone results in varying degrees of cellular injury: necrotic, delayed/indirect, and reversible injury. Various processes including apoptosis, ischemia-reperfusion, and immune response are responsible for delayed or indirect cellular injury (8).

Tumor necrosis immediately releases damage-associated molecular patterns (DAMPs, such as DNAs and heat shock proteins (HSPs)) into the extracellular matrix (9). Dendritic cells (DCs) phagocytize DAMPs and subsequently promote the expression of co-stimulatory CD80/86 molecules (10, 11), present antigens on major histocompatibility complex (MHC) molecules, and promote systemic immune response (12).

Necroptosis, also called programmed necrosis, is a newly recognized form of regulated cell death with morphological features of both apoptosis and necrosis (13, 14). It is activated by death receptors, which subsequently induce RIPK3 (serine/threonine kinase 3) and activate MLKL (mixed lineage kinase domain-like pseudokinase). This ultimately leads to cell swelling and rupture as well as the release of DAMPs, hence potentially immunogenic (13, 14). Necroptosis has been observed in post-IRE environment (15, 16) but has not been reported or studied yet in other ablation modalities.

In contrast, apoptosis is an intrinsic, orchestrated process of cell death, known to be less immunogenic and inflammatory (10) than necrosis. Apoptosis also induces release of antigens that can be picked up by dendritic cells, but they do not typically release DAMPs (12). Without phagocytizing DAMPs, DCs do not induce expression of CD80 and CD86 molecules (12). Without these co-stimulators, T cell anergy or even clonal deletion may occur, thereby suppressing the immune response (12).

The relative ratio between tumor necrosis and apoptosis was hypothesized to be a critical modulator in post-ablation immunogenicity (3). Different ablation protocols and parameters have been utilized to optimize this ratio, resulting in varying degrees of post-ablation immunogenic profiles (17, 18).

## Post-ablation inflammation: a double-edged sword

Ablative therapy decreases overall tumor burden and possibly leads to the reversal of immune suppression and unmasks a population of primed tumor-specific T cells that can induce protective immunity (19). Although detailed mechanism and downstream effect of the post-ablation immune response still remains elusive at large, there are many studies identifying effectors and mediators. Ablated tumors release and expose immune-potentiating tumor antigens as well as nuclear protein high mobility group B1 (HMGB1) and HSPs, which in turn induce antitumor immune response through activation of DCs (20). Studies have demonstrated tumor neoantigen released from the ablation bed yielded more effective dendritic cells in inducing CD4^+^ and CD8^+^ T cells (21). It also induces transient activation of myeloid dendritic cells (MDCs) associated with increased serum levels of TNF-α and IL-1β (22). In addition, the post-ablation environment has been shown to accelerate the migration of peripheral blood mononuclear cells (PBMC), activation of effector cells, and induction and secretion of cytokines, all of which may enhance the antitumor immune response (23).

It remains unknown how or if these post-ablation responses turn into clinically meaningful anti-tumor effects or abscopal effects. There have been anecdotal clinical case reports of the abscopal phenomenon after local ablation therapies (24–27) while the majority of post-ablation immune response remains clinically quiescent (1). Several cellular biomarkers including elevated lymphocyte-monocyte ratio (LMR) (28), decreased neutrophil-lymphocyte ratio (NLR) (29, 30) and increased cytotoxic natural killer (NK) cells (31) have been correlated with favorable post-ablation clinical outcomes.

On the contrary, there are many clinical reports with accelerated tumor progression after ablation therapy, questioning if the post-ablation environment may be in fact pro-oncogenic (32–35). There are studies showing that RFA is inferior to surgical resection due to a higher risk of recurrence and metastasis (36–39), with 5-year overall recurrence rates of 63.5% compared to 41.7% in surgical resection (40). Other studies have shown rapid tumor progression in some patients after RFA, which may be due to residual viable tumors after insufficient RFA (IRFA) (41–46). There are several suggested explanations for prooncogenic response in the post-ablation period. Rozenblum et al. showed that liver regeneration induced by RF ablation facilitates c-met/hepatocyte growth factor axis-dependent hepatocellular carcinoma (HCC) formation and blockage of IL-6 or c-met significantly reduced hepatocyte proliferation in animal models (35, 47). Another study by Kumar et al. showed that post-ablation STAT3 (signal transducer and activator of transcription 3) activation is linked to increased distant tumor stimulation in animal models (48). In addition, studies found factors such as epithelial-mesenchymal transitions (EMTs), increased number of cancer stem cells (CSCs), sustained local inflammation and hypoxic microenvironment, and heat shock response could explain rapid tumor progression after thermal ablation (49).

## Ablation modality-specific immunomodulation

While all ablation techniques share eventual tissue destruction or elimination as a common endpoint, the mechanisms by which tissue is manipulated to achieve that end point differs substantially, and the resultant immunological effects might be expected to be similarly discrepant. In general, RFA is historically the most thoroughly studied and serves as the archetypal hyperthermic ablation modality.

The immunomodulatory effects of RFA are a complex interplay of both adaptive immunity activation and immunosuppressive effects. In general, in response to tumor ablation, numerous pro-inflammatory physiological pathways are activated, resulting in the upregulation of signaling molecules including IL(interleukin)-1β, IL-1α, IL-6, IL-8, IL-18, and TNFα (tumor necrosis factor α) (22, 47, 50, 51). Simultaneously, immunosuppressive molecules including IL-10 and transforming growth factor-beta are also elevated, and immunosuppressive T regulatory cells are decreased, reflecting the nuanced relationship between ablation and immune response (51, 52). Additional classes of signaling molecules, including “danger signals,” typically released by necrotic cells and implicated in the process of antigen presentation, are also affected, with extracellular heat shock protein 70 (HSP70) and high morbidity group box-1 (HMGB-1) among those studied (53–56). Further complicating our understanding of this complex interplay is the apparent heterogeneity with which these individual mechanisms are employed in any given patient, as evidenced by conflicting findings of up or down-regulation of various cytokines and regulatory T cells (47, 52, 57). At least some of this heterogeneity may be attributable to technical differences across patients, including treatment efficacy, with pro-tumoral molecules, including TNF-alpha, significantly higher among patients with subsequent tumor recurrence (58).

When compared head-to-head to alternative modalities, cryoablation has been shown to have the highest degree of immunomodulatory effects (59–62). Indeed, the well-described phenomenon of “cryoshock” whereby a hyper-activated immunological response to tumoral antigens results in a potentially lethal systemic inflammatory response represents an extreme example (63–66). Mechanistically, the relatively robust immune response following cryoablation is attributed to preservation of tumoral antigens, released from the ablation core, where necrotic cell death results from osmotic shock or physical damage, allowing spillage and subsequent uptake of these antigens by antigen presenting cells. This stands in contrast to hyperthermic ablation which results in coagulative necrosis with relatively more destruction of cellular proteins, leaving fewer viable antigens on which to predicate an immune response.

Studies of microwave ablation demonstrate the modality to have relatively fewer immunomodulatory effects; for example, serum levels of inflammatory cytokines have been shown to be lower after MWA as compared to RFA or cryoablation (59, 67). Even so, clinical reports of abscopal effects have been reported, and benchtop research has demonstrated increased tumor-specific T cell populations in HCC, as well as increased B-cell/CD4+ interactions in breast cancer (67–70). When present, these immunomodulatory effects connote an improved prognosis (68). Furthermore, “mild” microwave ablation at 48 degrees celsius has been shown to increase apoptotic rate in an *in vitro* and *in vivo* osteosarcoma model, when added to transforming growth factor (TGF) beta-1 inhibitor and heat shock protein 90 (HSP90) inhibitor, suggesting the immunomodulatory effects of microwave ablation may become increasingly appreciable in the era of immunomodulatory medications (71).

HIFU employs focused ultrasound, resulting in repeated collapse and growth of microbubbles within the target tissue, leading to mechanical disruption and boiling, with hyperthermic ablation and histotripsy as the end result (72–74). Histotripsy, which refers to cellular disruption into a liquified acellular homogenate, represents an immunological unique feature of HIFU as compared to other hyperthermic modalities. For example, in a study comparing tumor cells heated to below 55°C relative to above 80°C, there was increased dendritic cell maturation in the lower temperature group, suggesting histotripsy as an important underlying driver of the immune response, at least when allowed to predominate over coagulative necrosis (75). Additional studies have demonstrated the abscopal effects of HIFU, including showing an immunological benefit to HIFU in a melanoma model and neuroblastoma model (59, 76, 77).

The immune effects after laser ablation are relatively less well understood, as compared to other ablative modalities. One study of 13 consecutive patients undergoing percutaneous liver laser ablation demonstrated postprocedural increases in IL-6 and Tumor Necrosis Factor (TNF) Receptor 1, but not TNF-alpha, IL-1 beta, or IL-10 (78). In a murine model of colorectal liver metastases, CD3+ T cell accumulation was noted at the site of laser ablation, as well as within remote sites of disease (79). In a study of 11 patients undergoing hepatic laser ablation of colorectal metastases, the cytolytic activity of CD3+, CD4+, and CD8+ T cells was seen to increase after ablation (80).

As a younger ablative modality, the immune effects of IRE are also less well understood than RFA or cryoablation, especially as its mechanism for cell destruction - introducing nanopores into the lipid bi-layers of cell membranes - is so different from previously studied modalities (81). Early research that has been done on the immune effects of IRE show minimal inflammatory or fibrotic margins in the ablation zone, increased peripheral T lymphocytes when compared to surgical resection, and greater CD3+ T lymphocytes when compared to cryoablation (81–83). Recently, IRE’s upregulation of IFNγ has been suggested as a potential co-therapeutic target for immune regulatory medications (84).

## Synergistic effect of ablation and immunotherapy

As tumor ablation has been shown to prime one’s immune system by releasing tumor neoantigens, numerous studies in recent years have investigated the combinatory effects of ablation and adjuvant immunotherapy with the hopes of demonstrating their synergistic effects (1) (**Table 1**). Extending beyond investigations of the mechanisms underlying the interactions between ablation and immunotherapeutic agent administration, several clinical trials and studies have been implemented in pursuit of confirming potential clinical advantages of combination therapy. Difficulties remain in directly correlating outcomes and post-treatment immune modulation, though there are many promising results.

**Table 1:** Summary of currently available clinical studies on ablation and immunotherapies.

Method of Immunomodulation	Study	Phase/Study Type	Immunomodulator(s)	Ablative Approach	Disease (No. subjects)	Impact of combination therapy
ICI	McAurthur et al, 2016	I, II	Ipilimumab (anti-CTLA-4)	Cryoablation	Breast cancer (19)	↑ IFN-γ, ↑ effector T cells, ↑ effector T cells/regulatory T cells ratio in peripheral blood
ICI	Duffy et al, 2016	I, II	Tremelimumab (anti-CTLA-4)	RFA or chemoablation	HCC (41)	Median time to disease progression 7.4mo, median OS 12.3mo, ↑ *in situ* CD8+ cells in treatment responders
ICI	Bäcklund and Freedman, 2017	Case Report	Pembrolizumab (anti-PD1)	MWA	CRC lung metastasis (1)	Complete response at 8mo
ICI	Soule et al, 2018	Case Report	Nivolumab (anti-PD1)	Cryoablation	RCC (1)	Decreased size and FDG uptake in osseous metastatic disease in 1mo
ICI	Agdashian et al, 2019	I, II	Tremelimumab (anti-CTLA-4)	Cryoablation or RFA or TACE	HCC (39)	↑ tumor-specific T cell activation with tremelimumab, no significant change in TCR clonality after combined therapy
ICI	Xie et al, 2019	I, II	Tremelimumab (anti-CTLA-4)	MWA	Biliary tract cancer (20)	Feasibility and safety of combination therapy demonstrated; median PFS 3.4mo, OS 6.0mo (comparable to 2^nd^-line chemotherapy)
ICI	Lyu et al, 2020	II	Nivolumab (anti-PD-1) or pembrolizumab (anti-PD-1)	RFA or MWA	HCC (50)	↑ efficacy and response rate, median time to progression 6.1mo, median PFS 5mo, median OS 16.9mo
ICI	Shen et al, 2020	Cohort	Pembrolizumab (anti-PD-1)	Cryoablation	Melanoma liver metastases (15)	Median PFS 4.0mo, median hepatic PFS 5.73mo, ↑ NK cells and ↓Treg cells 3 weeks after first treatment cycle in combined stage
ICI	Wang et al, 2021	Observational	Camrelizumab (anti-PD-1)	RFA	HCC (127)	↑ 1-year RFS, ↑ OS with combined therapy
Peptide	Si et al, 2009	I, II	Intratumoral GM-CSF	Cryoablation	Prostate cancer (12)	↑ tumor-specific effector T cells in peripheral blood
Peptide	Thakur et al, 2011	I, II	Intratumoral GM-CSF	Cryoablation	RCC (6)	↑ tumor-specific IFN-gamma and effector T cells, ↑ Th1/Th2 ratio in peripheral blood of treatment responders
Peptide	Sawada et al, 2016	II	GPC3-derived peptide vaccine	RFA or surgery	HCC (41)	↓ 1-year recurrence rates in patients with GPC3-positive tumor
Allogeneic immune cell transfer	Weng et al, 2008		CIK	RFA + TACE	HCC (85)	↓1-year and 18mo recurrence rates with combined therapy
Allogeneic immune cell transfer	Ma et al, 2010	I	RetroNectin-activated killer cells	RFA	HCC (7)	Feasibility and safety of combination therapy, no recurrence at 7mo
Allogeneic immune cell transfer	Zhou et al, 2011	I	Immature DCs + CIK + CTL	MWA	HCC (10)	↑ effector T cells, ↓ regulatory T cells in peripheral blood
Allogeneic immune cell transfer	Huang et al, 2013	Observational	CIK	RFA + TACE	HCC (85)	↑ median OS, ↑ median PFS
Allogeneic immune cell transfer	Niu et al, 2013	Observational	GM-CSF-stimulated DCs	Cryoablation	Pancreatic cancer (106)	↑ median OS with combined therapy
Allogeneic immune cell transfer	Niu et al, 2013	Observational	GM-CSF-stimulated DCs	Cryoablation	HCC (45)	↑ median OS with combined therapy
Allogeneic immune cell transfer	Cui et al, 2014	I	CIK + NK cells + γδT cells	RFA	HCC (62)	↑ OS, ↑ PFS with combined therapy
Allogeneic immune cell transfer	Li et al, 2014	Meta-analysis	CIK	TACE +/- RFA	HCC (189)	↑ 1-year RFS, ↑ 1- and 2-year OS with combined therapy
Allogeneic immune cell transfer	Lee et al, 2015	III	CIK	RFA or PEIT or surgical resection	HCC (115)	↑ OS, ↑ tumor-specific survival rate with combined therapy
Allogeneic immune cell transfer	Yu et al, 2015	II	CIK	MWA	HCC (14)	↑ absolute lymphocyte count, ↑ certain cytotoxic T cell subsets, ↓ negative regulatory or helper T cell subsets, ↑ serum albumin
Allogeneic immune cell transfer	Lee et al, 2017	II	DCs	RFA	HCC (156)	↓ recurrence rate with treatment with DC immunotherapy combined with standard treatment other than RFA
Allogeneic immune cell transfer	Liang et al, 2017	I, II	Stimulated allogenic NK cells	Cryoablation	Breast cancer (48)	↑ IFN-gamma, ↑ effector T cells in peripheral blood with combinatory treatment
Allogeneic immune cell transfer	Lin et al, 2017	I, II	Stimulated allogenic NK cells	Cryoablation	Renal cell cancer (60)	↑ Th1 cytokines, ↑ effector T cells in peripheral blood with combinatory treatment
Allogeneic immune cell transfer	Lin et al, 2017	I, II	Stimulated allogenic NK cells	Cryoablation	NSCLC (60)	↑ Th1 cytokines, ↑ effector T cells in peripheral blood with combinatory treatment
Allogeneic immune cell transfer	Yang et al, 2019	I, II	Stimulated allogenic NK cells	IRE	Primary liver cancer (40)	↑ OS, ↑ PFS, ↑ Th1 cytokines, ↑ effector T cells in peripheral blood with combinatory treatment
Allogeneic immune cell transfer	Huang et al, 2020	Observational	CIK	MWA + TACE	HCC (100)	↑ OS, ↑ PFS with combined therapy
Allogeneic immune cell transfer	Kitahara et al, 2020	I, II	OK432-stimulated DCs	RFA	HCC, HCV-related (30)	↑ 5-year RFS in patients with significantly increased TAA-specific T cell response
Allogeneic immune cell transfer	Ji et al, 2021	Observational	CIK	RFA + TACE	HCC (116)	↑ CD4+ T cells, ↑ CD4+/CD8+ ratio, ↑ Tregs, ↑ NK cells, ↓ CD8 T cells, ↑ quality of life scores and ↑ 5-year OS with combination therapy

↑, increase; ↓, decrease; CIK, cytokine-induced killer; CTL, cytotoxic lymphocyte; DC, dendritic cell; GM-CSF, granulocyte-macrophage colony-stimulating factor; GPC3, glypican-3; HCC, hepatocellular carcinoma; HCV, hepatitis C virus; ICI, immune checkpoint inhibitor; IRE, irreversible electroporation; MWA, microwave ablation; NK, natural killer; OS, overall survival; PEIT, percutaneous ethanol injection therapy; PFS, progression-free survival; RCC, renal cell carcinoma; RFA, radiofrequency ablation; RFS, recurrence-free survival; TACE, transarterial chemoembolization.

Combinations of ablation with immune checkpoint inhibitors (ICI) in preclinical and clinical studies have offered favorable prospects. PD-1 blockade with nivolumab or pembrolizumab has been of particular interest for potential synergy with thermal ablative techniques, owing to evidence of a theoretical influence of PD-L1/PD-1 expression on RFA-induced antitumor immunity (85). One proof-of-concept clinical trial demonstrated that subtotal RFA or MWA may augment the tumor-directed immune response stimulated by these agents to increase the overall response rate and improve median overall survival (OS) in patients with HCC (86). Likewise, survival advantages have been suggested with camrelizumab (anti-PD-1) combined with RFA, shown in a propensity score matching analysis of 127 patients to improve 1-year recurrence-free survival (RFS) (87). In another pilot study, 15 patients with melanoma metastases to the liver who underwent cryoablation and pembrolizumab experienced persistently increased serum IL-6 levels immediately after cryoablation (88). At three weeks following treatment, there was a significant increase in Natural Killer (NK) cells and a decrease in regulatory T (Treg) cells. Case reports have furthermore reported successful treatment of colorectal metastases to the lung with the combination of MWA and pembrolizumab (89), as well as of metastatic clear cell RCC with the combination of cryoablation and nivolumab (90).

The use of ablation in conjunction with CTLA-4 blockade, especially by means of tremelimumab administration, may generate favorable immune processes in patients as well. In a pilot study of 12 BCLC stage C patients with metastatic HCC that did not respond to sorafenib treatment, Duffy and colleagues revealed that treatment with RFA and chemoablation therapy combined with tremelimumab led to favorable objective treatment responses, with median TTP (time to progression) of 7.4 months and median OS of 10.1 months (91), as well as a decreased volume of and increased active CD8+ cytotoxic lymphocyte (CTL) infiltration into distant lesions. The authors then went on to evaluate the efficacy of combining tremelimumab with RFA, cryoablation, or Transarterial Chemoembolization (TACE) in treating HCC (92) and with MWA in treating advanced biliary tract cancer (BTC) (93). The feasibility and safety of combination therapy were successfully demonstrated in both instances and data revealed early evidence that tremelimumab with MWA may produce differential systemic immune responses in BTC and HCC patients (93). However, all 39 HCC and 20 BTC patients studied underwent tumor ablation or TACE rendered it impossible to address the question of whether locoregional therapy enhanced the effects of ICI (92). Additionally, a pilot study assessing the feasibility of neoadjuvant treatment with another anti-CTLA-4 antibody (ipilimumab) and cryoablation in patients with operable breast cancer suggested tolerability of the combination without delay of surgical intervention, along with increased activated proliferating peripheral T cells following combination therapy compared to either treatment alone (94).

There have been promising studies looking at harnessing the potentiation of the antigen presentation process in combination with ablation therapies. Two studies investigating treatment with cryoablation and GM-CSF injection in patients with metastatic RCC (95) and prostate cancer (96) showed tumor-specific CTL response in peripheral blood. Meanwhile, the adoptive transfer of stimulated dendritic cells (DCs) themselves has also proven a candidate for use with ablation. Niu and colleagues conducted a retrospective review of 106 patients with metastatic pancreatic cancer (97), and the later at 45 patients with metastatic HCC (98), demonstrating that median survival and overall survival, respectively, were significantly improved when treated with a combination of cryoablation and adoptive transfer of GM-CSF-stimulated DCs than with cryoablation, immunotherapy, or chemotherapy alone (97, 98). A randomized phase I/II trial of 30 HCC patients investigated autologous dendritic cell tumor injection versus RFA combined with OK432-stimulated DC tumor injection (99). The study found that 5-year RFS rates of patients with significantly increased tumor-associated antigen (TAA)-specific T cell responses were much higher than those of other patients, aligned with results of previous studies showing that RFA increases the TAA-related T cell response in murine models of HCC (100). However, in another phase II study, adjuvant therapy with a TAA-pulsed DC vaccine prolonged RFS of patients with complete remission in non-RFA groups compared with those in the RFA-treated group (101), leaving the question of optimal DC treatment combinations yet to be clarified. The neoadjuvant and adjuvant potential of tumor antigen vaccines is also being explored. For instance, based on the finding that RFA tended to induce a significantly greater GPC3-specific CTL response in patients with GPC3-overexpressing HCC than TACE or surgical resection (102), the antitumor efficacy and resulting GPC3-specific CTL response following administration of a GPC3 peptide vaccine was established (103), and a phase II study of the combination of the vaccine with RFA or resection demonstrated improved 1-year recurrence rates (104), though differences between patients receiving surgical versus ablative local treatments remain unclear.

In addition to DCs, other cell types have also demonstrated signs of antitumor response when administered by adoptive transfer and hence have been interrogated for synergistic interactions with ablation. Researchers have long sought to capture the malignant cell-recognizing capability of natural killer (NK) cells, innate immune effector lymphocytes that utilize self-ligand recognition to issue rapid destructive methods against stressed cells (105), in cancer immunotherapy. In an early pilot clinical study of NK cell transfer and cryoablation in 48 patients with treatment-resistant breast cancer, effector T cells and Th1-type cytokines were increased in the peripheral blood of patients who received the combined treatment, but progression-free survival (PFS) was not significantly augmented in this group compared to those who only underwent cryoablation (106). Similar peripheral immune cell trends were seen in two prospective studies looking at this combination in 60 patients with metastatic RCC (107) and in 60 patients with non-small cell lung cancer (108), though data regarding survival were not reported. Clinical effects of NK cell immunotherapy were also studied in conjunction with IRE in primary liver cancer patients and were suggested to improve survival and elicit greater immune responses than IRE without immunotherapy (109).

Cytokine-induced killer (CIK) cells are non-major histocompatibility complex (MHC) restricted cytotoxic lymphocytes that display high proliferative and cytolytic activity (110), obtained by *in vitro* co-culture of human PBMCs and multiple cytokines (111). Existing data have offered substantial support for the possible benefits of CIK cell therapy in treating cancers in combination with other immunomodulatory modalities, with clinical investigations focusing mainly on implications in HCC treatment. A phase I study in ten HCC patients with chronic hepatitis B treated with MWA along with adoptive immunotherapy involving DC, CTL, and CIK infusion demonstrated the safety of this sequential combination of therapies and consequent signs of ameliorated immune suppression on peripheral blood analyses (112). Similar alterations to peripheral lymphocyte populations were seen in a prospective phase II study of 14 HCC patients treated with MWA with CIK cells alone (113), and this combination of therapies further been demonstrated in a study of 43 HCC patients to improve median OS and PFS when combined with TACE compared to MWA and TACE without immunotherapy (114). CIK cells administered in conjunction with RFA have furthermore been implicated in improving HCC treatment. OS and PFS were found to be significantly increased in a study of 30 patients with HCC treated with RFA followed by a combined infusion of CIK cells, NK cells, and γδT cells (115). In turn, when applied following a combination of RFA and TACE, CIK therapy has been revealed in meta-analysis data to be associated with higher 1-year RFS and 1- and 2-year OS than RFA plus TACE alone (116), and retrospective analyses of HCC patients treated with this triple therapy (n = 85 and n = 58, respectively) demonstrated significantly longer OS and PFS (117) as well as elevated peripheral Tregs and NK cells, lower CD8+ T cell levels, and higher general functioning scores than those who did not receive immunotherapy (118). In a prospective randomized trial, 45 HCC patients who underwent autologous CIK cell transfusion combined with RFA and TACE experienced no serious side effects and demonstrated reduced 1-year recurrence rates than those who received only RFA and TACE (9% versus 30%) (119). One multicenter, randomized phase III clinical study enrolled 230 HCC patients who had undergone surgical resection, RFA, or percutaneous ethanol injection (PEI), who were then randomly assigned to receive or not receive CIK cell infusions. The group assigned to undergo combination therapy with CIK cells had a median PFS of 44 months and had significantly longer OS and tumor-specific survival rates than the control group (101). Finally, one single-arm prospective study of 7 patients with HCC treated with RFA followed by autologous T lymphocytes stimulated with a combination of immobilized RetroNectin and anti-CD3 monoclonal antibody, which has been suggested to induce greater T cell expansion than other known methods (120, 121), demonstrated the safety of this treatment approach as well as improvements in circulating lymphocyte profiles (122), opening up avenues for the investigation of additional forms of adjuvant adoptive immunotherapy and their role in ablative cancer treatment.

## Ongoing trials and future direction

As of September 2022, 60 clinical trials studying combinations of various ablation and immunotherapy are registered to be planned or underway at clinicaltrials.gov (**Table 2**). Among these are a few notable large, multicenter phase III trials, including EMERALD-2 and CA2099DX. Most of the clinical trial landscape, however, remains dominated by outcome-based studies of small sample sizes and great variability in treatment parameters and protocols, reflecting rapid recent advancements in both interventional oncology and immuno-oncology. Therefore, in addition to optimizing combination treatment regimens, a detailed understanding of the mechanisms and key players of synergistic interaction should be prioritized for future studies. For example, identifications of biomarkers from post-ablation tumor microenvironments will help us to select the most effective, personalized therapeutic strategy among multiple available modalities and possible combinations with immunotherapy agents.

**Table 2:** Summary of ongoing trials of locoregional interventions and immunotherapy.

Disease	Ablative/Locoregional Therapy	Immunotherapy	Additional Intervention	Phase/ Study Type	No. patients	End Date	Trial No.
Adenocarcinoma, unspecified	RF-EMB	Intratumoral anti-PD-1 + anti-CTLA-4 + GM-CSF		Observational	27	**06/2020**	NCT03695835
Breast cancer	Cryoablation	Ipilimumab (anti-CTLA-4) + Nivolumab (anti-PD-1)		Pilot	5 (est)	06/2023	NCT02833233
Breast cancer	Cryoablation	NK cells		I, II	30	**07/2019**	NCT02844335
Breast cancer	MWA	Camrelizumab (anti-PD-1)	Breast-conserving surgery or radical mastectomy	II	60 (est)	12/2022	NCT04805736
Cervical cancer, recurrent	Cryoablation	NK cells		I, II	30	**07/2019**	NCT02849340
Cholangiocarcinoma, intrahepatic	Cryoablation	Camrelizumab (anti-PD-1)		II	25 (est)	05/2023	NCT04299581
CRC	Cryoablation	AlloStim^®^ alloantigens		II	12	09/2018	NCT02380443
CRC	RFA	Pembrolizumab (anti-PD-1)	Radiation therapy	II	34	04/2023	NCT02437071
CRC, liver metastases	RFA	Durvalumab (anti-PD-1) + Tremelimumab (anti-CTLA-4)	SBRT	II	22	**02/2022**	NCT03101475
CRC, liver metastases	RFA	GM-CSF + TLR or NOD2 agonist	Mifamurtide (L-MTP-PE)	I	12 (est)	09/2024	NCT04062721
CRC, liver metastases	RFA	Regorafenib (multi-kinase inhibitor) + Toripalimab (anti-PD-1)		II	32 (est)	06/2024	NCT05485909
CRC, liver/pulmonary metastases	MWA	Camrelizumab (anti-PD-1)	Chemotherapy	II	37 (est)	06/2025	NCT04888806
Hepatocellular Carcinoma/Biliary Tract Cancer	Cryoablation or RFA or TACE	Durvalumab (anti-PD-1) + Tremelimumab (anti-CTLA-4)		I, II	54	12/2022	NCT02821754
Hepatocellular Carcinoma	IRE	Nivolumab (anti-PD-1)		II	43	11/2023	NCT03630640
Hepatocellular carcinoma	MWA	Intravenous CIK cells + abdominal cavity DC-CIK and CTL		II, III	40	03/2021	NCT02851784
Hepatocellular Carcinoma	MWA	Neoantigen-based DC vaccine		I	24 (est)	12/2020	NCT03674073
Hepatocellular Carcinoma	MWA + TACE	Sintilimab (anti-PD1)		I	45 (est)	09/2022	NCT04220944
Hepatocellular Carcinoma	MWA or RFA or brachytherapy +/- TACE	Pembrolizumab (anti-PD-1)		II	30 (est)	05/2024	NCT03753659
Hepatocellular Carcinoma	MWA or RFA	Tislelizumab (anti-PD-1)		II	30 (est)	12/2023	NCT04652440
Hepatocellular Carcinoma	Thermal ablation	Toripalimab (anti-PD-1)		II	116	07/2024	NCT05240404
Hepatocellular Carcinoma	MWA or RFA	Toripalimab (anti-PD-1)		I, II	145	06/2023	NCT03864211
Hepatocellular Carcinoma	Ablation (unspecified) or surgical resection	Durvalumab (anti-PD-1) +/- Bevacizumab		III	908	05/2024	NCT03847428
Hepatocellular Carcinoma	Ablation (unspecified) or surgical resection	Nivolumab (anti-PD-1)		III	545	12/2025	NCT03383458
Hepatocellular Carcinoma	RFA or TACE or PEIT	CIK cells		I, II	55 (est)	06/2019	NCT03124498
Hepatocellular Carcinoma	RFA	highly purified CTLs		III	210 (est)	01/2022	NCT02678013
Hepatocellular Carcinoma	RFA	Tislelizumab or Sintilimab (anti-PD-1) + Lenvatinib (TKI) or Bevacizumab (anti-VEGF)		NR	160 (est)	10/2023	NCT05277675
Hepatocellular Carcinoma	RFA or surgical resection	Autologous NK cells		II	140 (est)	04/2019	NCT02725996
Laryngeal cancer	Cryoablation	NK cells		I, II	30	**07/2019**	NCT02849314
Lung cancer/Melanoma	Cryoablation	ICI (unspecified)	CNB	II	20 (est)	03/2025	NCT03290677
Lung cancer, multiple	MWA	Camrelizumab (Anti-PD1)		II	146 (est)	09/2027	NCT05053802
Malignant neoplasm, unspecified	Cryoablation	Standard of care immunotherapy for tumor type	CNB	NR	25 (est)	08/2023	NCT04150939
Melanoma, cutaneous	Cryoablation	Intratumoral DCs + Pembrolizumab (anti-PD-1)	Apheresis	I, II	7	10/2022	NCT03325101
Melanoma, cutaneous	Cryoablation	*In situ* Ipilimumab (anti-CTLA-4) + intravenous Nivolumab (anti-PD-1)		I, II	19	**03/2022**	NCT03949153
Melanoma, cutaneous	RFT + Cryoablation or RFA	Sargramostim (GM-CSF)	CNB	I	11	**05/2018**	NCT00568763
Melanoma, liver metastases	Cryoablation	Tislelizumab (anti-PD-1) + Lenvatinib (TKI)		II	25 (est)	08/2024	NCT05406466
Non-Hodgkin lymphoma	Cryoablation	Pembrolizumab (anti-PD-1) + DC vaccine	Pneumococcal 13-valent conjugate vaccine	I, II	11	07/2023	NCT03035331
NSCLC, metastatic	Cryoablation	NK cells		I, II	30	**07/2019**	NCT02843815
NSCLC	MWA	Camrelizumab (Anti-PD1)	Chemotherapy	I, II	35 (est)	09/2025	NCT05532527
NSCLC	MWA	Pembrolizumab (anti-PD-1)		III	100 (est)	11/2021	NCT03769129
Ovarian cancer	Cryoablation	NK cells		I, II	30	**07/2019**	NCT02849353
Pancreatic cancer	IRE	Nivolumab (anti-PD-1)		II	10 (est)	04/2026	NCT03080974
Pancreatic cancer	IRE	Nivolumab (anti-PD-1)		II	12 (est)	09/2026	NCT05435053
Pancreatic cancer	IRE	Nivolumab (anti-PD-1) + CpG (TLR ligand)		I	18 (est)	10/2022	NCT04612530
Pancreatic cancer	MWA	Durvalumab (anti-PD-L1) + Tremelimumab (anti-CTLA-4)		II	20 (est)	12/2023	NCT04156087
Pancreatic cancer, single metastatic liver lesion	IRE	Pembrolizumab (anti-PD-1)		II	8	12/2025	NCT04835402
Pharyngeal cancer, recurrent	Cryoablation	NK cells		I, II	30	**07/2019**	NCT02849327
Primary liver tumor, transplanted liver	Cryoablation	NK cells		I, II	30	**07/2019**	NCT02849015
Primary liver tumor, recurrent	IRE	NK cells		I, II	20	**07/2019**	NCT03008343
Prostate cancer	Cryoablation	Ipilimumab (anti-CTLA-4) + Intratumoral DCs	Cyclophosphamide	I	18	**8/2019**	NCT02423928
Prostate cancer	Cryoablation	Pembrolizumab (anti-PD-1)	Degarelix (GnRH antagonist)	Pilot	12	**11/2017**	NCT02489357
Prostate cancer	Cryoablation	Sargramostim (GM-CSF)		I	19	**12/2020**	NCT02250014
Renal cell carcinoma	Cryoablation	NK cells		I, II	30	**07/2019**	NCT02843607
Renal cell carcinoma	Cryoablation	Tremelimumab (anti-CTLA-4)	Surgical resection, biopsy	I	29	**06/2022**	NCT02626130
Renal cell carcinoma	RFA	Interferon-α or sunitinib (TKI)		I, II	114	**01/2011**	NCT00891475
Soft tissue sarcoma, recurrent	Cryoablation	NK cells		I, II	30	**07/2019**	NCT02849366
Solid tumors, advanced, unspecified	Laser ablation	Intratumoral IP-001		I, II	39 (est)	12/2023	NCT03993678
Solid tumors, unspecified	RFA	anti-PD-1 + GM-CSF + iNeo-Vac-P01 (neoantigen peptides)		I	30 (est)	08/2025	NCT04864379
Tongue cancer	Cryoablation	NK cells		I, II	30	**07/2019**	NCT02849379
Unspecified	Cryoablation	Intratumoral AlloStim^®^ alloantigens		I, II	50 (est)	**05/2011**	NCT00861107

Bolded end dates are actual dates of study completion.

CIK, cytokine-induced killer; CNB, core needle biopsy; CTL, cytotoxic lymphocyte; DC, dendritic cell; GM-CSF, granulocyte-macrophage colony-stimulating factor; GnRH, gonadotropin releasing hormone; ICI, immune checkpoint inhibitor; IFN, interferon; IP-001, 1% N-dihydro-galacto-chitosan; IRE, irreversible electroporation; L-MTP-PE, liposomal muramyl tripeptide phosphatidyl ethanolamine; mCRC, metastatic colorectal cancer; MWA, microwave ablation; NK, natural killer; NOD2, nucleotide binding oligomerization domain containing 2; NR, not reported; PEIT, percutaneous ethanol injection therapy; RFA, radiofrequency ablation; RF-EMB, radiofrequency electrical membrane breakdown; RFT, radiofrequency therapy; SBRT, stereotactic body radiation therapy; TACE, transarterial chemoembolization; TKI, tyrosine kinase inhibitor; TLR, toll-like receptor; VEGF, vascular endothelial growth factor.

## Conclusion

Percutaneous ablation treatment offered by interventional oncology is not only effective in local tumor control but also capable of releasing tumor neoantigens in situ. Although successful in priming the immune system, this rarely turns into clinical benefits of local and systemic tumor control due to intrinsic negative immune modulation of the tumor microenvironment. A combination of ablation and immunotherapy has shown preliminary but promising results of synergistic effect without significantly increased risk profiles. Synergistic interaction between tumor ablation and immunotherapy will further our understandings of cancer generation and treatment, which will greatly benefit scientists and patients together.

## Author contributions

DK contributed to conception and oversaw the entire review. NK, JY, AT, JL and DK performed literature reviews and wrote sections of the manuscript. NK and JY created tables. NK created the graphics. All authors contributed to manuscript revision, read, and approved the submitted version.
